# Coordination complexes of chromium(0) with a series of 1,3-diphenyl-6-aryl­fulvenes

**DOI:** 10.1107/S2056989018010794

**Published:** 2018-08-10

**Authors:** Andrew J. Peloquin, Madelyn B. Smith, Bryce J. O’Connell, Kamran B. Ghiassi, Gary J. Balaich, Scott T. Iacono

**Affiliations:** a Department of Chemistry & Chemistry Research Center, United States Air Force Academy, Colorado Springs, CO 80840, USA; b Aerospace Systems Directorate, Air Force Research Laboratory, Edwards AFB, CA 93524, USA

**Keywords:** crystal structure, penta­fulvene, chromium, piano stool, π-π inter­actions

## Abstract

The synthesis and structural properties of a series of chromium tricarbonyl ‘piano-stool’ complexes bearing substituted penta­fulvene ligands were studied. The complexes, tricarbon­yl(1,3,6-tri­phenyl­fulvene)chromium(0) benz­ene hemisolvate, [Cr(C_24_H_18_)(CO)_3_]·0.5C_6_H_6_ (**I**), tricarbon­yl[1,3-di­phen­yl-6-(3-vinyl­phen­yl)fulvene]chromium(0), [Cr(C_26_H_20_)(CO)_3_] (**II**), and tricarbon­yl[1,3-diphenyl-6-(pyren-1-yl)fulvene]chromium(0), [Cr(C_34_H_22_)(CO)_3_] (**III**), each have a distorted octa­hedral geometry, with the fulvene coordinated in a π–η^2^:π–η^2:^π–η^2^ fashion. Significant deviation of the exocyclic fulvene double bond from the cyclo­penta­diene plane accompanies coordination. Evidence of non-covalent π–π inter­actions was observed in both (**I**) and (**III**), with centroid-to-centroid distances ranging from 3.330 (8) to 3.494 (8) Å.

## Abstract 2

The synthesis and structural properties of a series of chromium tricarbonyl ‘piano-stool’ complexes bearing substituted penta­fulvene ligands were studied. Significant deviation of the exocyclic fulvene double bond from the cyclo­penta­diene plane accompanies coordination. Evidence of non-covalent π-π inter­actions was observed.

## Chemical context   

Penta­fulvenes have been investigated because of their unique cross-conjugated electronic system. Despite the ability to tune the fulvene’s steric and electronic properties through substitution, their coordination chemistry remains relatively unexplored. As a result of their electronic structure, fulvenes display a variety of coordination behaviors with metals, ranging from π–η^2^, typically with late transition metals (O’Conner *et al.*, 1997 ▸), to π–η^5^:σ–η^1^,which is more common with early transition metals (Ebert *et al.*, 2014 ▸). Metal–fulvene complexes have been probed for hydro­amination catalysis (Janssen *et al.*, 2010 ▸), olefin metathesis (Erker *et al.*, 1991 ▸), and cytotoxicity (Deally *et al.*, 2011 ▸). Reduction to yield a cyclo­penta­diene ligand (Gómez-Ruiz *et al.*, 2005 ▸) or reductive coupling to form *ansa* bis-cyclo­penta­diene ligands (Adas & Balaich, 2018 ▸) are the most common examples of fulvene reaction chemistry. Herein, we report on the synthesis and structural properties of a series of chromium(0) complexes formed from 1,3-diphenyl-6-aryl fulvenes.
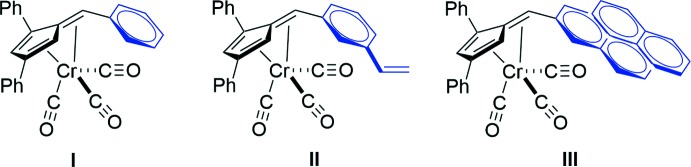


## Structural commentary   

**Figure 1:**
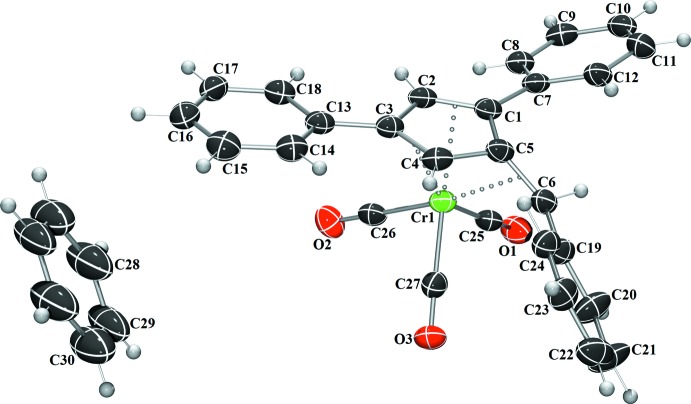
The mol­ecular structure of **I**. Displacement ellipsoids are shown at the 50% probability level.

**Figure 2:**
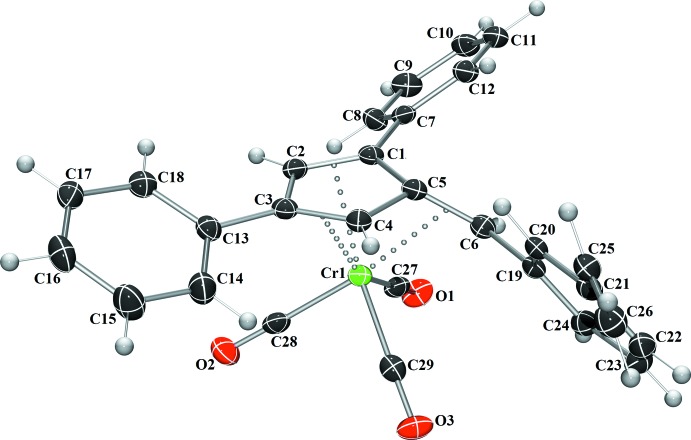
The mol­ecular structure of **II**. Displacement ellipsoids are shown at the 50% probability level.

**Figure 3:**
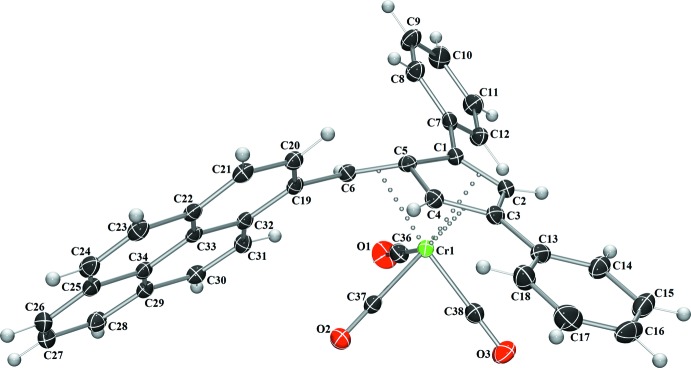
The mol­ecular structure of **III**. Displacement ellipsoids are shown at the 50% probability level.

Complex **I** crystallizes in the monoclinic space group *P*2_1_/*n*, (Fig. 1 ▸), complex **II** in the monoclinic space group *P*2_1_/*c* (Fig. 2 ▸), and complex **III** in the triclinic space group *P* 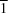 (Fig. 3 ▸), each with one mol­ecule per asymmetric unit. A benzene mol­ecule was found co-crystallized and located on an inversion center in the structure of **I**. In each complex, the coord­ination geometry around the chromium(0) atom is distorted octa­hedral, with the midpoints of the three formal fulvene double bonds and the three carbonyl carbons describing the six verticies of the octa­hedra. Analysis of the fulvene bond lengths when compared to the previously reported uncomplexed fulvenes reveals nearly unchanged C—C single bonds (C1—C5, C4—C5, and C2—C3) with slight elongation of the C=C double bonds (C1=C2, C3=C4, and C5=C6) (Table 1 ▸). This double-bond elongation is typical upon π-coordination to a metal atom. Based upon the alternating short and long bond distances, the coordination mode of the fulvene to the chromium atom is best described as π–η^2^:π–η^2:^π–η^2^ in nature. Additionally, the coordination of the fulvene exocyclic double bond (C5=C6) results in the bending of this bond from the cyclo­penta­diene plane by 33.22 (18) to 34.2 (3)°. This is in agreement with a previously reported chromium complex with 6,6-di­methyl­fulvene (Konietzny *et al.*, 2010 ▸).

## Supra­molecular features   

**Figure 4:**
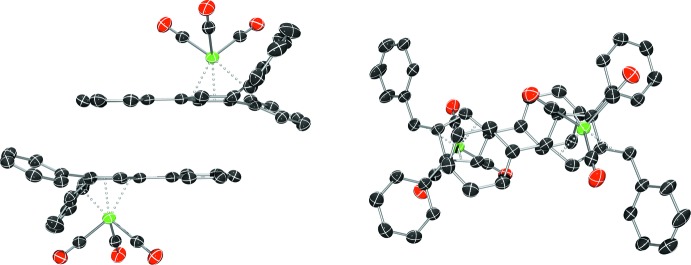
The π–π stacking arrangement of **I**, viewed in the plane (left) and normal to the place (right) of the cyclo­penta­diene-phenyl rings. Displacement ellipsoids are shown at the 50% probability level. Hydrogen atoms have been omitted for clarity.

**Figure 5:**
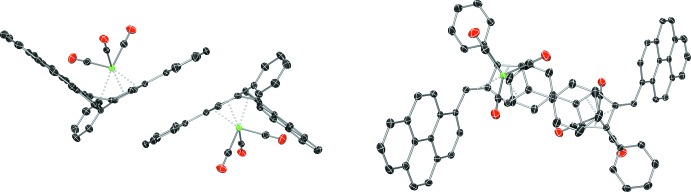
The π–π stacking arrangement of **III**, viewed in the plane (left) and normal to the place (right) of the cyclo­penta­diene-phenyl rings. Displacement ellipsoids are shown at the 50% probability level. Hydrogen atoms have been omitted for clarity.

Evidence for π–π inter­actions in the solid state is observed in **I** and **III**. In both **I** and **III**, the mol­ecules are arranged in layers in which the π system composed of the cyclo­penta­diene core (head) of the fulvene and the 3-phenyl substituent (tail) adopt a head-to-tail (Peterson *et al.*, 1999 ▸) π–π stacked arrangement. The inter­planar contact distance is 3.420 (17) Å in **I** (Fig. 4 ▸) and 3.330 (8) Å in and **III** (Fig. 5 ▸), both well within the distance expected for a non-covalent π–π inter­ation (Gruber *et al.*, 2008 ▸). In **I**, the centroid of each cyclo­penta­diene ring is slipped by 0.470 (17) Å end-to-end and 1.505 (17) Å edge-to-edge with respect to the opposing 3-phenyl substituent centroid. This results in a near perfect alignment of the fulvene C2 atom over the centroid of the opposing phenyl ring, with angles betwen the cyclo­penta­diene-phenyl ring normal and the C2 to phenyl ring centroid vector of only 2.05 (2)° end-to-end and 5.85 (3)° edge-to-edge. In complex **III**, the centroid of each cyclo­penta­diene ring is by slipped 0.286 (8) Å end-to-end and 0.761 (7) Å edge-to-edge with respect to the opposing 3-phenyl substituent centroid. Again, the C2 fulvene atom is brought into near perfect alignment over the centroid of the opposing phenyl ring, with angles betwen the cyclo­penta­diene-phenyl ring normal and the C2 to phenyl ring centroid vector of 7.67 (9)° end-to-end and 6.16 (9)° edge-to-edge.

**Figure 6:**
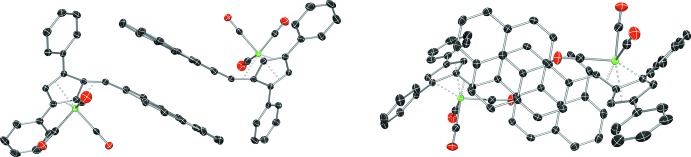
The π–π stacking arrangement of **III**, viewed in the plane (left) and normal to the place (right) of the pyrene rings. Displacement ellipsoids are shown at the 50% probability level. Hydrogen atoms have been omitted for clarity.

Further non-covalent π–π inter­actions are observed in **III** between the pyrene units. The inter­planar contact distance is 3.494 (8) Å (Fig. 6 ▸), with the centroids of the pyrene rings of π-stacked dimers slipped by 2.352 (7) Å in the end-to-end direction when viewed down the normals of the pyrene rings (Fig. 6 ▸). The ring centroids remain aligned in the edge-to-edge direction. The carbon atoms of opposing pyrene rings are brought close to perfect alignment with carbon atoms in the opposing ring system, slipped by one half a ring width. This is in contrast to the stacking arrangement observed in the uncomplexed fulvene, where the overlap is inter­mediate between full carbon-to-carbon alignment and carbon-to-ring-centroid alignment (Peloquin *et al.* 2012 ▸).

## Synthesis and crystallization   

The fulvenes 1,3,6-tri­phenyl­fulvene, 1,3-diphenyl-6-(3-vinyl­phen­yl)fulvene, and 1,3-diphenyl-6-(1-pyrene)fulvene were prepared in accordance with literature procedures (Peloquin *et al.*, 2012 ▸; Godman *et al.*, 2016 ▸).

**(1,3,6-Tri­phenyl­fulvene)tri­carbonyl­chromium(0) (I).** A solution of 1,3,6-tri­phenyl­fulvene (0.518 g, 1.69 mmol) in THF (10 mL) was added to a stirred suspension of Cr(CO)_3_(MeCN)_3_ (0.499 g, 1.93 mmol) in THF (15 mL) under N_2_. The solution quickly turned from pale yellow to dark red. The reaction mixture was allowed to stir at room temperature for 24 h before removal of the solvent *in vacuo*. The residue was dissolved in diethyl ether (100 mL), filtered under ambient conditions, and the solvent removed *in vacuo*. Crystals suitable for single-crystal X-ray diffraction were obtained by dissolving the crude product in benzene and layering with pentane.

**{1,3-Diphenyl-6-(3-vinyl­phen­yl)fulvene}tri­carbonyl­chromium(0) (II).** 1,3-Diphenyl-6-(3-vinyl­phen­yl)fulvene (0.637 g, 1.92 mmol) and Cr(CO)_3_(MeCN)_3_ (0.494 g, 1.92 mmol) were used employing the procedure outlined for the preparation of **I**. Crystals suitable for single-crystal X-ray diffraction were obtained by dissolving the crude product in benzene and layering with pentane.

**{1,3-Diphenyl-6-(1-pyrene)fulvene}tri­carbonyl­chromium(0) (III).** 1,3-Diphenyl-6-(1-pyrene)fulvene (0.603 g, 1.40 mmol) and Cr(CO)_3_(MeCN)_3_ (0.401 g, 1.54 mmol) were used employing the procedure outlined for the preparation of **I**. Crystals suitable for single-crystal X-ray diffraction were obtained by vapor diffusion of diethyl ether into a chloro­form solution of the crude product.

## Refinement   

Crystal data, data collection and structure refinement details are summarized in Table 2 ▸. All H atoms were positioned with idealized geometry and refined using a riding model, with C–H = 0.95 Å, and with *U*_iso_(H) = 1.2 *U*_eq_(C). In **I**, an outlier (101) was omitted in the last cycles of refinement.

## Supplementary Material

Crystal structure: contains datablock(s) global, I, II, III. DOI: 10.1107/S2056989018010794/rz5242sup1.cif

Structure factors: contains datablock(s) I. DOI: 10.1107/S2056989018010794/rz5242Isup2.hkl

Structure factors: contains datablock(s) II. DOI: 10.1107/S2056989018010794/rz5242IIsup3.hkl

Structure factors: contains datablock(s) III. DOI: 10.1107/S2056989018010794/rz5242IIIsup4.hkl

CCDC references: 1858307, 1858306, 1858305

Additional supporting information:  crystallographic information; 3D view; checkCIF report

## Figures and Tables

**Table 1:** Selected geometric parameters (Å, °) for **I**, **II**, and **III** and the corresponding fulvenes

	**I**	1,3,6-tri­phenylfulvene*^*a*^*	**II**	1,3-diphenyl-6-(3-vinylphen­yl)fulvene*^*b*^*	**III**	1,3-diphenyl-6-(1-pyrene)fulvene*^*a*^*
C1—C5/C4—C5	1.468 (5)/1.444 (6)	1.4860 (15)/1.4599 (16)	1.468 (4)/1.449 (4)	1.484 (2)/1.462 (2)	1.463 (2)/1.454 (2)	1.488 (2)/1.459 (2)
C1=C2/C3=C4	1.401 (5)/1.416 (5)	1.3553 (16)/1.3603 (16)	1.403 (4)/1.406 (4)	1.357 (2)/1.360 (2)	1.406 (2)/1.412 (2)	1.353 (2)/1.363 (2)
C2—C3	1.435 (5)	1.4660 (16)	1.444 (4)	1.469 (2)	1.443 (2)	1.467 (2)
C5=C6	1.394 (5)	1.3540 (16)	1.412 (4)	1.353 (2)	1.413 (2)	1.357 (2)
Cr1—C1/Cr1—Cr4	2.181 (4)/2.158 (4)		2.186 (2)/2.162 (3)		2.1809 (16)/2.1569 (16)	
Cr1—C2/Cr1—C3	2.237 (4)/2.265 (4)		2.243 (3)/2.251 (3)		2.2353 (16)/2.2477 (16)	
Cr1—C5	2.063 (4)		2.066 (3)		2.0704 (16)	
Cr1—C6	2.427 (4)		2.448 (3)		2.4428 (16)	
Fulvene*^*c*^*-(1-phen­yl)*^*d*^*	25.2 (2)	28.11 (6)	35.60 (14)	37.27 (9)	32.28 (9)	42.12 (7)
Fulvene*^*c*^*–(3-phen­yl)^*d*^	5.2 (2)	20.38 (6)	26.77 (14)	21.26 (9)	1.91 (10)	22.81 (7)
Fulvene–C6*^*e*^*	34.2 (3)	8.90 (9)	33.22 (18)	5.62 (12)	34.08 (14)	5.50 (10)

Notes: (*a*) Peloquin *et al.* (2012 ▸); (*b*) Shurdha *et al.*, 2014 ▸; (*c*) plane defined by atoms C1–C5; (*d*) plane defined by atoms of the specific phenyl ring substituent; (*e*) angle between the C5—C6 bond and the plane defined by the atoms C1–C5

**Table 2:** Experimental details

	**I**	**II**	**III**
Crystal data			
Chemical formula	[Cr(C_24_H_18_)(CO)_3_]·0.5C_6_H_6_	[Cr(C_26_H_20_)(CO)_3_]	[Cr(C_34_H_22_)(CO)_3_]
*M*_r_	481.47	468.45	566.54
Crystal system, space group	Monoclinic, *P*2_1_/*n*	Monoclinic, *P*2_1_/*c*	Triclinic, *P* 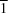
Temperature (K)	100	100	100
*a*, *b*, *c* (Å)	15.838 (5), 7.675 (2), 19.485 (6)	15.624 (4), 8.149 (2), 17.396 (4)	8.6342 (12), 9.4070 (13), 17.379 (3)
α, β, γ (°)	90, 99.335 (4), 90	90, 90.706 (3), 90	82.988 (2), 86.340 (2), 70.992 (2)
*V* (Å^3^)	2337.2 (13)	2214.7 (10)	1324.2 (3)
*Z*	4	4	2
Radiation type	Mo *K*α	Mo *K*α	Mo *K*α
μ (mm^−1^)	0.52	0.55	0.47
Crystal size (mm)	0.31 × 0.26 × 0.23	0.21 × 0.17 × 0.13	0.27 × 0.20 × 0.11

Data collection			
Diffractometer	Bruker SMART APEX CCD	Bruker SMART APEX CCD	Bruker SMART APEX CCD
Absorption correction	Multi-scan (*SADABS*; Bruker, 2017 ▸)	Multi-scan (*SADABS*; Bruker, 2017 ▸)	Multi-scan (*SADABS*; Bruker, 2017 ▸)
*T*_min_, *T*_max_	0.77, 0.89	0.82, 0.93	0.88, 0.95
No. of measured, independent and observed [*I* > 2σ(*I*)] reflections	38380, 4302, 2584	36033, 4086, 3183	25620, 5892, 5241
*R*_int_	0.152	0.075	0.029
(sin θ/λ)_max_ (Å^−1^)	0.604	0.604	0.644

Refinement			
*R*[*F* ^2^ > 2σ(*F* ^2^)], *wR*(*F* ^2^), *S*	0.057, 0.141, 1.03	0.042, 0.109, 1.08	0.036, 0.098, 1.05
No. of reflections	4302	4086	5892
No. of parameters	307	298	370
H-atom treatment	H-atom parameters constrained	H-atom parameters constrained	H-atom parameters constrained
Δρ_max_, Δρ_min_ (e Å^−3^)	0.56, −0.33	0.38, −0.40	0.48, −0.38

Computer programs: *APEX3* and *SAINT* (Bruker, 2017 ▸), *SHELXT* (Sheldrick, 2015*a* ▸), *SHELXL* (Sheldrick, 2015*b* ▸), *ORTEP-3* for Windows (Farrugia, 2012 ▸), *Mercury* (Macrae *et al.*, 2008 ▸) and *publCIF* (Westrip, 2010 ▸).

## supplementary crystallographic information

### Tricarbonyl(1,3,6-triphenylfulvene)chromium(0) benzene hemisolvate (I) Crystal data

**Table:**

[Cr(C_24_H_18_)(CO)_3_]·0.5C_6_H_6_	*F*(000) = 996
*M**_r_* = 481.47	*D*_x_ = 1.368 Mg m^−^^3^
Monoclinic, *P*2_1_/*n*	Mo *K*α radiation, λ = 0.71073 Å
*a* = 15.838 (5) Å	Cell parameters from 1393 reflections
*b* = 7.675 (2) Å	θ = 2.6–16.4°
*c* = 19.485 (6) Å	µ = 0.52 mm^−^^1^
β = 99.335 (4)°	*T* = 100 K
*V* = 2337.2 (13) Å^3^	Block, translucent red
*Z* = 4	0.31 × 0.26 × 0.23 mm

### Tricarbonyl(1,3,6-triphenylfulvene)chromium(0) benzene hemisolvate (I) Data collection

**Table:**

Bruker SMART APEX CCD diffractometer	4302 independent reflections
Radiation source: fine focus sealed tube	2584 reflections with *I* > 2σ(*I*)
Graphite monochromator	*R*_int_ = 0.152
Detector resolution: 8.3333 pixels mm^-1^	θ_max_ = 25.4°, θ_min_ = 2.6°
ω Scans scans	*h* = −19→19
Absorption correction: multi-scan (SADABS; Bruker, 2017)	*k* = −9→9
*T*_min_ = 0.77, *T*_max_ = 0.89	*l* = −23→23
38380 measured reflections	

### Tricarbonyl(1,3,6-triphenylfulvene)chromium(0) benzene hemisolvate (I) Refinement

**Table:**

Refinement on *F*^2^	0 restraints
Least-squares matrix: full	Hydrogen site location: inferred from neighbouring sites
*R*[*F*^2^ > 2σ(*F*^2^)] = 0.057	H-atom parameters constrained
*wR*(*F*^2^) = 0.141	*w* = 1/[σ^2^(*F*_o_^2^) + (0.040*P*)^2^ + 3.0871*P*] where *P* = (*F*_o_^2^ + 2*F*_c_^2^)/3
*S* = 1.03	(Δ/σ)_max_ = 0.001
4302 reflections	Δρ_max_ = 0.56 e Å^−^^3^
307 parameters	Δρ_min_ = −0.33 e Å^−^^3^

### Tricarbonyl(1,3,6-triphenylfulvene)chromium(0) benzene hemisolvate (I) Special details

**Table:**

Geometry. All esds (except the esd in the dihedral angle between two l.s. planes) are estimated using the full covariance matrix. The cell esds are taken into account individually in the estimation of esds in distances, angles and torsion angles; correlations between esds in cell parameters are only used when they are defined by crystal symmetry. An approximate (isotropic) treatment of cell esds is used for estimating esds involving l.s. planes.

### Tricarbonyl(1,3,6-triphenylfulvene)chromium(0) benzene hemisolvate (I) Fractional atomic coordinates and isotropic or equivalent isotropic displacement parameters (Å^2^)

**Table:**

	*x*	*y*	*z*	*U*_iso_*/*U*_eq_	
Cr1	0.55485 (4)	0.83445 (9)	0.67800 (3)	0.0316 (2)	
O1	0.46466 (19)	1.1237 (4)	0.74002 (16)	0.0466 (8)	
C1	0.4347 (2)	0.6949 (5)	0.6473 (2)	0.0308 (10)	
O3	0.72286 (18)	0.8603 (4)	0.77558 (15)	0.0499 (9)	
C2	0.4702 (2)	0.7175 (5)	0.5866 (2)	0.0311 (10)	
H2	0.443934	0.780309	0.546767	0.037*	
O2	0.61821 (18)	1.1061 (4)	0.58690 (16)	0.0458 (8)	
C3	0.5518 (2)	0.6325 (5)	0.5929 (2)	0.0302 (10)	
C5	0.4984 (2)	0.6008 (5)	0.6969 (2)	0.0320 (10)	
C4	0.5681 (3)	0.5589 (5)	0.6603 (2)	0.0341 (10)	
H4	0.617232	0.492504	0.678694	0.041*	
C6	0.5122 (3)	0.6478 (5)	0.7669 (2)	0.0331 (10)	
H6	0.477061	0.737704	0.780389	0.040*	
C19	0.5764 (3)	0.5709 (5)	0.8222 (2)	0.0337 (10)	
C7	0.3487 (2)	0.7486 (5)	0.6586 (2)	0.0315 (10)	
C24	0.6142 (3)	0.4109 (6)	0.8145 (2)	0.0360 (10)	
H24	0.599003	0.348061	0.772305	0.043*	
C8	0.3037 (3)	0.8793 (5)	0.6189 (2)	0.0344 (10)	
H8	0.330681	0.943199	0.586706	0.041*	
C23	0.6739 (3)	0.3405 (6)	0.8671 (2)	0.0409 (11)	
H23	0.699419	0.230736	0.861009	0.049*	
C9	0.2202 (3)	0.9172 (6)	0.6257 (2)	0.0403 (11)	
H9	0.190155	1.006501	0.598104	0.048*	
C22	0.6957 (3)	0.4313 (6)	0.9283 (2)	0.0494 (13)	
H22	0.736811	0.384661	0.964613	0.059*	
C10	0.1801 (3)	0.8260 (6)	0.6725 (2)	0.0405 (11)	
H10	0.122579	0.852126	0.676887	0.049*	
C21	0.6583 (3)	0.5886 (7)	0.9369 (2)	0.0574 (15)	
H21	0.673189	0.650829	0.979328	0.069*	
C11	0.2244 (3)	0.6956 (6)	0.7131 (2)	0.0432 (12)	
H11	0.197495	0.632131	0.745376	0.052*	
C20	0.5987 (3)	0.6574 (6)	0.8841 (2)	0.0496 (13)	
H20	0.572839	0.766468	0.890725	0.059*	
C12	0.3079 (2)	0.6599 (6)	0.7057 (2)	0.0360 (10)	
H12	0.338215	0.571829	0.733865	0.043*	
C13	0.6061 (3)	0.6217 (5)	0.5387 (2)	0.0335 (10)	
C18	0.5840 (3)	0.7074 (5)	0.4753 (2)	0.0370 (11)	
H18	0.532863	0.774272	0.466765	0.044*	
C17	0.6358 (3)	0.6959 (6)	0.4247 (2)	0.0388 (11)	
H17	0.619517	0.753794	0.381526	0.047*	
C16	0.7106 (3)	0.6018 (6)	0.4363 (2)	0.0402 (11)	
H16	0.746757	0.597396	0.401973	0.048*	
C15	0.7325 (3)	0.5137 (6)	0.4984 (2)	0.0415 (11)	
H15	0.784015	0.448031	0.506678	0.050*	
C14	0.6803 (3)	0.5203 (5)	0.5485 (2)	0.0354 (10)	
H14	0.694940	0.455092	0.590207	0.043*	
C25	0.5009 (3)	1.0135 (6)	0.7170 (2)	0.0345 (10)	
C26	0.5945 (2)	1.0009 (6)	0.6223 (2)	0.0330 (10)	
C29	0.9972 (4)	0.6345 (9)	0.5459 (4)	0.084 (2)	
H29	0.995074	0.728012	0.577493	0.100*	
C30	1.0533 (4)	0.5006 (9)	0.5636 (4)	0.088 (2)	
H30	1.089990	0.500655	0.607343	0.105*	
C28	0.9440 (4)	0.6357 (8)	0.4829 (4)	0.087 (2)	
H28	0.905248	0.729633	0.471121	0.105*	
C27	0.6586 (3)	0.8500 (5)	0.7375 (2)	0.0358 (10)	

### Tricarbonyl(1,3,6-triphenylfulvene)chromium(0) benzene hemisolvate (I) Atomic displacement parameters (Å^2^)

**Table:**

	*U*^11^	*U*^22^	*U*^33^	*U*^12^	*U*^13^	*U*^23^
Cr1	0.0298 (4)	0.0332 (4)	0.0296 (4)	−0.0036 (3)	−0.0018 (3)	0.0012 (3)
O1	0.0386 (18)	0.047 (2)	0.055 (2)	−0.0015 (15)	0.0079 (15)	−0.0126 (16)
C1	0.031 (2)	0.028 (2)	0.031 (2)	−0.0069 (18)	−0.0016 (18)	0.0017 (19)
O3	0.0355 (18)	0.075 (2)	0.0353 (18)	−0.0107 (16)	−0.0070 (15)	0.0026 (17)
C2	0.031 (2)	0.029 (2)	0.030 (2)	−0.0066 (18)	−0.0058 (18)	0.0028 (18)
O2	0.0397 (18)	0.0429 (19)	0.056 (2)	0.0003 (15)	0.0096 (16)	0.0134 (17)
C3	0.031 (2)	0.026 (2)	0.030 (2)	−0.0053 (18)	−0.0016 (18)	0.0008 (18)
C5	0.029 (2)	0.032 (2)	0.032 (2)	−0.0052 (18)	−0.0029 (19)	0.0067 (19)
C4	0.030 (2)	0.035 (2)	0.034 (2)	−0.0001 (19)	−0.0036 (19)	−0.004 (2)
C6	0.033 (2)	0.032 (2)	0.032 (2)	−0.0024 (19)	0.0003 (18)	0.003 (2)
C19	0.034 (2)	0.036 (3)	0.029 (2)	−0.002 (2)	−0.0013 (19)	0.006 (2)
C7	0.029 (2)	0.035 (2)	0.028 (2)	−0.0055 (19)	−0.0046 (18)	−0.0023 (19)
C24	0.040 (3)	0.037 (3)	0.030 (2)	−0.003 (2)	0.003 (2)	0.004 (2)
C8	0.034 (2)	0.033 (2)	0.033 (2)	−0.0033 (19)	−0.0024 (19)	0.0005 (19)
C23	0.044 (3)	0.042 (3)	0.036 (3)	0.002 (2)	0.004 (2)	0.011 (2)
C9	0.039 (3)	0.041 (3)	0.037 (3)	0.001 (2)	−0.007 (2)	−0.006 (2)
C22	0.046 (3)	0.050 (3)	0.046 (3)	−0.011 (2)	−0.010 (2)	0.014 (3)
C10	0.029 (2)	0.052 (3)	0.040 (3)	−0.007 (2)	0.002 (2)	−0.013 (2)
C21	0.086 (4)	0.049 (3)	0.028 (3)	−0.008 (3)	−0.016 (3)	0.002 (2)
C11	0.031 (2)	0.055 (3)	0.042 (3)	−0.008 (2)	0.001 (2)	0.002 (2)
C20	0.074 (3)	0.040 (3)	0.030 (3)	0.003 (3)	−0.007 (2)	0.003 (2)
C12	0.031 (2)	0.042 (3)	0.033 (2)	−0.005 (2)	−0.0032 (18)	0.004 (2)
C13	0.034 (2)	0.029 (2)	0.035 (2)	−0.0082 (19)	−0.0001 (19)	−0.0026 (19)
C18	0.035 (2)	0.034 (3)	0.040 (3)	−0.0043 (19)	−0.001 (2)	0.000 (2)
C17	0.044 (3)	0.042 (3)	0.029 (2)	−0.011 (2)	0.003 (2)	−0.001 (2)
C16	0.044 (3)	0.040 (3)	0.037 (3)	−0.006 (2)	0.007 (2)	−0.009 (2)
C15	0.043 (3)	0.030 (2)	0.050 (3)	−0.001 (2)	0.005 (2)	−0.004 (2)
C14	0.038 (3)	0.030 (2)	0.037 (3)	0.000 (2)	0.000 (2)	0.000 (2)
C25	0.027 (2)	0.041 (3)	0.033 (2)	−0.005 (2)	−0.0018 (19)	−0.003 (2)
C26	0.026 (2)	0.035 (3)	0.036 (2)	0.0001 (19)	−0.0020 (19)	−0.003 (2)
C29	0.047 (4)	0.091 (5)	0.118 (6)	−0.025 (4)	0.029 (4)	−0.064 (4)
C30	0.060 (4)	0.099 (5)	0.105 (5)	−0.020 (4)	0.020 (4)	−0.055 (4)
C28	0.050 (4)	0.085 (5)	0.128 (6)	−0.011 (3)	0.020 (4)	−0.051 (4)
C27	0.039 (3)	0.038 (3)	0.030 (2)	−0.005 (2)	0.006 (2)	0.001 (2)

### Tricarbonyl(1,3,6-triphenylfulvene)chromium(0) benzene hemisolvate (I) Geometric parameters (Å, º)

**Table:**

Cr1—C25	1.845 (5)	C23—C22	1.376 (6)
Cr1—C26	1.851 (5)	C23—H23	0.9500
Cr1—C27	1.855 (4)	C9—C10	1.383 (6)
Cr1—C5	2.063 (4)	C9—H9	0.9500
Cr1—C4	2.158 (4)	C22—C21	1.368 (7)
Cr1—C1	2.181 (4)	C22—H22	0.9500
Cr1—C2	2.237 (4)	C10—C11	1.392 (6)
Cr1—C3	2.265 (4)	C10—H10	0.9500
Cr1—C6	2.427 (4)	C21—C20	1.384 (6)
O1—C25	1.153 (5)	C21—H21	0.9500
C1—C2	1.401 (5)	C11—C12	1.380 (6)
C1—C5	1.468 (5)	C11—H11	0.9500
C1—C7	1.474 (5)	C20—H20	0.9500
O3—C27	1.161 (4)	C12—H12	0.9500
C2—C3	1.435 (5)	C13—C18	1.394 (6)
C2—H2	0.9500	C13—C14	1.396 (5)
O2—C26	1.162 (5)	C18—C17	1.384 (6)
C3—C4	1.416 (5)	C18—H18	0.9500
C3—C13	1.467 (6)	C17—C16	1.374 (6)
C5—C6	1.394 (5)	C17—H17	0.9500
C5—C4	1.444 (6)	C16—C15	1.380 (6)
C4—H4	0.9500	C16—H16	0.9500
C6—C19	1.479 (5)	C15—C14	1.380 (6)
C6—H6	0.9500	C15—H15	0.9500
C19—C20	1.372 (6)	C14—H14	0.9500
C19—C24	1.386 (6)	C29—C30	1.366 (9)
C7—C12	1.384 (5)	C29—C28	1.371 (9)
C7—C8	1.391 (5)	C29—H29	0.9500
C24—C23	1.386 (5)	C30—C28^i^	1.390 (8)
C24—H24	0.9500	C30—H30	0.9500
C8—C9	1.381 (6)	C28—H28	0.9500
C8—H8	0.9500		
			
C25—Cr1—C26	87.21 (19)	C5—C6—H6	116.9
C25—Cr1—C27	96.76 (18)	C19—C6—H6	116.9
C26—Cr1—C27	88.37 (17)	Cr1—C6—H6	90.7
C25—Cr1—C5	109.34 (18)	C20—C19—C24	118.0 (4)
C26—Cr1—C5	154.46 (17)	C20—C19—C6	119.7 (4)
C27—Cr1—C5	108.07 (17)	C24—C19—C6	122.2 (4)
C25—Cr1—C4	149.20 (18)	C12—C7—C8	118.2 (4)
C26—Cr1—C4	122.09 (17)	C12—C7—C1	120.3 (4)
C27—Cr1—C4	93.62 (17)	C8—C7—C1	121.4 (4)
C5—Cr1—C4	39.92 (16)	C19—C24—C23	121.4 (4)
C25—Cr1—C1	92.12 (17)	C19—C24—H24	119.3
C26—Cr1—C1	122.83 (16)	C23—C24—H24	119.3
C27—Cr1—C1	148.00 (17)	C9—C8—C7	120.6 (4)
C5—Cr1—C1	40.35 (14)	C9—C8—H8	119.7
C4—Cr1—C1	65.06 (15)	C7—C8—H8	119.7
C25—Cr1—C2	111.65 (16)	C22—C23—C24	119.3 (4)
C26—Cr1—C2	91.51 (16)	C22—C23—H23	120.4
C27—Cr1—C2	151.55 (17)	C24—C23—H23	120.4
C5—Cr1—C2	64.65 (15)	C8—C9—C10	120.4 (4)
C4—Cr1—C2	62.67 (15)	C8—C9—H9	119.8
C1—Cr1—C2	36.96 (14)	C10—C9—H9	119.8
C25—Cr1—C3	148.72 (16)	C21—C22—C23	120.1 (4)
C26—Cr1—C3	90.65 (16)	C21—C22—H22	120.0
C27—Cr1—C3	114.39 (17)	C23—C22—H22	120.0
C5—Cr1—C3	64.99 (15)	C9—C10—C11	119.7 (4)
C4—Cr1—C3	37.23 (14)	C9—C10—H10	120.2
C1—Cr1—C3	63.20 (15)	C11—C10—H10	120.2
C2—Cr1—C3	37.17 (14)	C22—C21—C20	120.1 (4)
C25—Cr1—C6	86.85 (17)	C22—C21—H21	119.9
C26—Cr1—C6	170.52 (16)	C20—C21—H21	119.9
C27—Cr1—C6	85.01 (16)	C12—C11—C10	119.2 (4)
C5—Cr1—C6	35.01 (14)	C12—C11—H11	120.4
C4—Cr1—C6	65.23 (15)	C10—C11—H11	120.4
C1—Cr1—C6	64.80 (14)	C19—C20—C21	121.1 (5)
C2—Cr1—C6	97.55 (14)	C19—C20—H20	119.4
C3—Cr1—C6	98.25 (14)	C21—C20—H20	119.4
C2—C1—C5	106.8 (3)	C11—C12—C7	121.9 (4)
C2—C1—C7	126.8 (3)	C11—C12—H12	119.0
C5—C1—C7	126.3 (4)	C7—C12—H12	119.0
C2—C1—Cr1	73.7 (2)	C18—C13—C14	118.0 (4)
C5—C1—Cr1	65.5 (2)	C18—C13—C3	121.2 (4)
C7—C1—Cr1	127.4 (3)	C14—C13—C3	120.8 (4)
C1—C2—C3	110.5 (3)	C17—C18—C13	120.5 (4)
C1—C2—Cr1	69.4 (2)	C17—C18—H18	119.7
C3—C2—Cr1	72.5 (2)	C13—C18—H18	119.7
C1—C2—H2	124.7	C16—C17—C18	120.7 (4)
C3—C2—H2	124.7	C16—C17—H17	119.6
Cr1—C2—H2	125.0	C18—C17—H17	119.6
C4—C3—C2	106.7 (4)	C17—C16—C15	119.3 (4)
C4—C3—C13	127.2 (4)	C17—C16—H16	120.3
C2—C3—C13	126.1 (4)	C15—C16—H16	120.3
C4—C3—Cr1	67.3 (2)	C16—C15—C14	120.5 (4)
C2—C3—Cr1	70.3 (2)	C16—C15—H15	119.7
C13—C3—Cr1	128.3 (3)	C14—C15—H15	119.7
C6—C5—C4	121.9 (4)	C15—C14—C13	120.8 (4)
C6—C5—C1	119.9 (4)	C15—C14—H14	119.6
C4—C5—C1	106.5 (3)	C13—C14—H14	119.6
C6—C5—Cr1	86.9 (3)	O1—C25—Cr1	177.7 (4)
C4—C5—Cr1	73.6 (2)	O2—C26—Cr1	179.0 (4)
C1—C5—Cr1	74.2 (2)	C30—C29—C28	120.8 (6)
C3—C4—C5	109.3 (4)	C30—C29—H29	119.6
C3—C4—Cr1	75.5 (2)	C28—C29—H29	119.6
C5—C4—Cr1	66.5 (2)	C29—C30—C28^i^	119.3 (6)
C3—C4—H4	125.4	C29—C30—H30	120.4
C5—C4—H4	125.4	C28^i^—C30—H30	120.4
Cr1—C4—H4	124.1	C29—C28—C30^i^	119.9 (6)
C5—C6—C19	126.2 (4)	C29—C28—H28	120.0
C5—C6—Cr1	58.1 (2)	C30^i^—C28—H28	120.0
C19—C6—Cr1	121.2 (3)	O3—C27—Cr1	178.9 (4)
			
C5—C1—C2—C3	3.7 (4)	C5—C1—C7—C12	−24.8 (6)
C7—C1—C2—C3	−174.1 (4)	Cr1—C1—C7—C12	−109.9 (4)
Cr1—C1—C2—C3	61.0 (3)	C2—C1—C7—C8	−22.3 (6)
C5—C1—C2—Cr1	−57.3 (3)	C5—C1—C7—C8	160.3 (4)
C7—C1—C2—Cr1	124.9 (4)	Cr1—C1—C7—C8	75.2 (5)
C1—C2—C3—C4	−1.5 (4)	C20—C19—C24—C23	−0.9 (6)
Cr1—C2—C3—C4	57.6 (3)	C6—C19—C24—C23	−179.6 (4)
C1—C2—C3—C13	177.2 (4)	C12—C7—C8—C9	−0.9 (6)
Cr1—C2—C3—C13	−123.6 (4)	C1—C7—C8—C9	174.0 (4)
C1—C2—C3—Cr1	−59.2 (3)	C19—C24—C23—C22	0.2 (6)
C2—C1—C5—C6	139.4 (4)	C7—C8—C9—C10	0.2 (6)
C7—C1—C5—C6	−42.8 (6)	C24—C23—C22—C21	0.4 (7)
Cr1—C1—C5—C6	76.8 (3)	C8—C9—C10—C11	0.2 (6)
C2—C1—C5—C4	−4.4 (4)	C23—C22—C21—C20	−0.3 (8)
C7—C1—C5—C4	173.4 (4)	C9—C10—C11—C12	0.0 (6)
Cr1—C1—C5—C4	−67.0 (3)	C24—C19—C20—C21	1.0 (7)
C2—C1—C5—Cr1	62.6 (3)	C6—C19—C20—C21	179.7 (4)
C7—C1—C5—Cr1	−119.6 (4)	C22—C21—C20—C19	−0.4 (8)
C2—C3—C4—C5	−1.4 (4)	C10—C11—C12—C7	−0.7 (6)
C13—C3—C4—C5	179.9 (4)	C8—C7—C12—C11	1.2 (6)
Cr1—C3—C4—C5	58.2 (3)	C1—C7—C12—C11	−173.8 (4)
C2—C3—C4—Cr1	−59.6 (3)	C4—C3—C13—C18	−177.0 (4)
C13—C3—C4—Cr1	121.7 (4)	C2—C3—C13—C18	4.5 (6)
C6—C5—C4—C3	−139.3 (4)	Cr1—C3—C13—C18	−88.1 (4)
C1—C5—C4—C3	3.6 (4)	C4—C3—C13—C14	5.0 (6)
Cr1—C5—C4—C3	−63.8 (3)	C2—C3—C13—C14	−173.5 (4)
C6—C5—C4—Cr1	−75.5 (4)	Cr1—C3—C13—C14	94.0 (4)
C1—C5—C4—Cr1	67.4 (3)	C14—C13—C18—C17	−2.0 (6)
C4—C5—C6—C19	−39.0 (6)	C3—C13—C18—C17	−179.9 (4)
C1—C5—C6—C19	−177.2 (4)	C13—C18—C17—C16	−0.8 (6)
Cr1—C5—C6—C19	−107.5 (4)	C18—C17—C16—C15	2.0 (6)
C4—C5—C6—Cr1	68.5 (3)	C17—C16—C15—C14	−0.2 (6)
C1—C5—C6—Cr1	−69.7 (3)	C16—C15—C14—C13	−2.6 (6)
C5—C6—C19—C20	162.9 (4)	C18—C13—C14—C15	3.7 (6)
Cr1—C6—C19—C20	91.8 (5)	C3—C13—C14—C15	−178.3 (4)
C5—C6—C19—C24	−18.4 (6)	C28—C29—C30—C28^i^	0.0 (11)
Cr1—C6—C19—C24	−89.6 (4)	C30—C29—C28—C30^i^	0.0 (11)
C2—C1—C7—C12	152.5 (4)		

Symmetry code: (i) −*x*+2, −*y*+1, −*z*+1.

### Tricarbonyl[1,3-diphenyl-6-(3-vinylphenyl)fulvene]chromium(0) (II) Crystal data

**Table:**

[Cr(C_26_H_20_)(CO)_3_]	*F*(000) = 968
*M**_r_* = 468.45	*D*_x_ = 1.405 Mg m^−^^3^
Monoclinic, *P*2_1_/*c*	Mo *K*α radiation, λ = 0.71073 Å
*a* = 15.624 (4) Å	Cell parameters from 4824 reflections
*b* = 8.149 (2) Å	θ = 2.3–23.2°
*c* = 17.396 (4) Å	µ = 0.55 mm^−^^1^
β = 90.706 (3)°	*T* = 100 K
*V* = 2214.7 (10) Å^3^	Rectangular prism, translucent red
*Z* = 4	0.21 × 0.17 × 0.13 mm

### Tricarbonyl[1,3-diphenyl-6-(3-vinylphenyl)fulvene]chromium(0) (II) Data collection

**Table:**

Bruker SMART APEX CCD diffractometer	4086 independent reflections
Radiation source: fine focus sealed tube	3183 reflections with *I* > 2σ(*I*)
Graphite monochromator	*R*_int_ = 0.075
Detector resolution: 8.3333 pixels mm^-1^	θ_max_ = 25.4°, θ_min_ = 2.3°
ω Scans scans	*h* = −18→18
Absorption correction: multi-scan (SADABS; Bruker, 2017)	*k* = −9→9
*T*_min_ = 0.82, *T*_max_ = 0.93	*l* = −20→21
36033 measured reflections	

### Tricarbonyl[1,3-diphenyl-6-(3-vinylphenyl)fulvene]chromium(0) (II) Refinement

**Table:**

Refinement on *F*^2^	0 restraints
Least-squares matrix: full	Hydrogen site location: inferred from neighbouring sites
*R*[*F*^2^ > 2σ(*F*^2^)] = 0.042	H-atom parameters constrained
*wR*(*F*^2^) = 0.109	*w* = 1/[σ^2^(*F*_o_^2^) + (0.0425*P*)^2^ + 2.3842*P*] where *P* = (*F*_o_^2^ + 2*F*_c_^2^)/3
*S* = 1.08	(Δ/σ)_max_ = 0.001
4086 reflections	Δρ_max_ = 0.38 e Å^−^^3^
298 parameters	Δρ_min_ = −0.40 e Å^−^^3^

### Tricarbonyl[1,3-diphenyl-6-(3-vinylphenyl)fulvene]chromium(0) (II) Special details

**Table:**

Geometry. All esds (except the esd in the dihedral angle between two l.s. planes) are estimated using the full covariance matrix. The cell esds are taken into account individually in the estimation of esds in distances, angles and torsion angles; correlations between esds in cell parameters are only used when they are defined by crystal symmetry. An approximate (isotropic) treatment of cell esds is used for estimating esds involving l.s. planes.

### Tricarbonyl[1,3-diphenyl-6-(3-vinylphenyl)fulvene]chromium(0) (II) Fractional atomic coordinates and isotropic or equivalent isotropic displacement parameters (Å^2^)

**Table:**

	*x*	*y*	*z*	*U*_iso_*/*U*_eq_	
Cr1	0.71754 (3)	0.55453 (5)	0.55201 (2)	0.01855 (14)	
O1	0.77319 (13)	0.2211 (2)	0.49712 (11)	0.0322 (5)	
O2	0.57333 (13)	0.3776 (3)	0.63056 (11)	0.0339 (5)	
O3	0.81643 (13)	0.5446 (2)	0.70222 (11)	0.0311 (5)	
C1	0.70342 (16)	0.6522 (3)	0.43552 (14)	0.0184 (6)	
C2	0.62308 (17)	0.6686 (3)	0.46989 (14)	0.0198 (6)	
H2	0.571608	0.618815	0.451982	0.024*	
C3	0.63077 (17)	0.7730 (3)	0.53669 (14)	0.0199 (6)	
C4	0.71706 (16)	0.8194 (3)	0.54475 (14)	0.0185 (5)	
H4	0.739888	0.890262	0.583221	0.022*	
C5	0.76525 (16)	0.7408 (3)	0.48435 (14)	0.0191 (6)	
C6	0.84732 (16)	0.6731 (3)	0.49748 (14)	0.0199 (6)	
H6	0.860447	0.572376	0.472776	0.024*	
C7	0.72497 (16)	0.5658 (3)	0.36351 (14)	0.0190 (6)	
C8	0.68446 (18)	0.4207 (3)	0.34157 (15)	0.0240 (6)	
H8	0.640621	0.375975	0.372586	0.029*	
C9	0.70800 (19)	0.3413 (3)	0.27441 (16)	0.0276 (6)	
H9	0.68021	0.242145	0.25985	0.033*	
C10	0.77164 (18)	0.4053 (3)	0.22852 (15)	0.0261 (6)	
H10	0.788344	0.349185	0.183254	0.031*	
C11	0.81060 (18)	0.5511 (3)	0.24891 (15)	0.0239 (6)	
H11	0.853305	0.596641	0.216883	0.029*	
C12	0.78793 (17)	0.6313 (3)	0.31563 (14)	0.0220 (6)	
H12	0.815219	0.73155	0.329164	0.026*	
C13	0.55946 (17)	0.8350 (3)	0.58454 (15)	0.0209 (6)	
C14	0.57508 (18)	0.8946 (4)	0.65845 (16)	0.0293 (7)	
H14	0.630363	0.882259	0.681052	0.035*	
C15	0.51123 (19)	0.9716 (4)	0.69936 (18)	0.0339 (7)	
H15	0.522841	1.011459	0.749736	0.041*	
C16	0.43034 (19)	0.9906 (4)	0.66703 (17)	0.0303 (7)	
H16	0.386859	1.0461	0.694503	0.036*	
C17	0.41326 (18)	0.9280 (3)	0.59421 (16)	0.0248 (6)	
H17	0.357593	0.938888	0.572201	0.03*	
C18	0.47721 (17)	0.8497 (3)	0.55348 (16)	0.0226 (6)	
H18	0.4648	0.805799	0.503999	0.027*	
C19	0.91401 (16)	0.7478 (3)	0.54682 (14)	0.0188 (6)	
C20	0.98062 (17)	0.6517 (3)	0.57762 (15)	0.0232 (6)	
H20	0.981283	0.536665	0.568732	0.028*	
C21	1.04558 (18)	0.7241 (4)	0.62104 (15)	0.0259 (6)	
H21	1.090964	0.658546	0.641018	0.031*	
C22	1.04451 (17)	0.8916 (3)	0.63539 (15)	0.0237 (6)	
H22	1.08886	0.939876	0.665604	0.028*	
C23	0.97856 (17)	0.9897 (3)	0.60567 (14)	0.0204 (6)	
C24	0.91515 (16)	0.9163 (3)	0.56008 (14)	0.0188 (6)	
H24	0.871717	0.983008	0.537557	0.023*	
C25	0.97278 (17)	1.1672 (3)	0.62114 (15)	0.0240 (6)	
H25	0.943549	1.232838	0.584089	0.029*	
C26	1.00490 (18)	1.2428 (4)	0.68233 (16)	0.0278 (6)	
H26A	1.034618	1.181813	0.720779	0.033*	
H26B	0.998252	1.358104	0.687801	0.033*	
C27	0.75267 (17)	0.3499 (3)	0.51848 (15)	0.0231 (6)	
C28	0.62897 (18)	0.4450 (3)	0.60100 (15)	0.0236 (6)	
C29	0.77775 (17)	0.5491 (3)	0.64492 (16)	0.0219 (6)	

### Tricarbonyl[1,3-diphenyl-6-(3-vinylphenyl)fulvene]chromium(0) (II) Atomic displacement parameters (Å^2^)

**Table:**

	*U*^11^	*U*^22^	*U*^33^	*U*^12^	*U*^13^	*U*^23^
Cr1	0.0202 (2)	0.0194 (2)	0.0160 (2)	−0.00053 (18)	−0.00066 (16)	0.00103 (17)
O1	0.0367 (12)	0.0254 (11)	0.0342 (12)	0.0040 (9)	−0.0062 (9)	−0.0035 (9)
O2	0.0306 (12)	0.0438 (13)	0.0276 (11)	−0.0087 (10)	0.0044 (9)	0.0051 (10)
O3	0.0394 (12)	0.0327 (12)	0.0209 (11)	−0.0011 (9)	−0.0108 (9)	0.0005 (9)
C1	0.0225 (14)	0.0162 (13)	0.0165 (13)	−0.0019 (11)	−0.0012 (10)	0.0043 (10)
C2	0.0200 (14)	0.0218 (14)	0.0174 (13)	−0.0008 (11)	−0.0020 (10)	0.0033 (11)
C3	0.0234 (14)	0.0190 (13)	0.0173 (13)	0.0017 (11)	0.0007 (11)	0.0043 (11)
C4	0.0203 (13)	0.0173 (13)	0.0177 (13)	0.0002 (11)	−0.0001 (10)	0.0014 (10)
C5	0.0219 (14)	0.0198 (14)	0.0158 (12)	−0.0010 (11)	0.0006 (10)	0.0024 (10)
C6	0.0224 (14)	0.0172 (13)	0.0202 (13)	−0.0014 (11)	0.0017 (11)	0.0017 (11)
C7	0.0206 (13)	0.0208 (14)	0.0154 (13)	0.0022 (11)	−0.0030 (10)	0.0028 (10)
C8	0.0246 (15)	0.0282 (15)	0.0192 (14)	−0.0044 (12)	0.0012 (11)	0.0026 (11)
C9	0.0377 (17)	0.0216 (15)	0.0235 (14)	−0.0030 (13)	−0.0032 (12)	−0.0014 (12)
C10	0.0326 (16)	0.0294 (16)	0.0164 (13)	0.0037 (13)	0.0000 (12)	−0.0015 (12)
C11	0.0254 (15)	0.0286 (15)	0.0178 (14)	0.0013 (12)	0.0005 (11)	0.0032 (11)
C12	0.0241 (14)	0.0230 (14)	0.0190 (13)	−0.0013 (11)	−0.0003 (11)	0.0024 (11)
C13	0.0213 (14)	0.0198 (14)	0.0216 (14)	−0.0018 (11)	0.0029 (11)	0.0025 (11)
C14	0.0230 (15)	0.0387 (17)	0.0264 (15)	0.0004 (13)	0.0018 (12)	−0.0034 (13)
C15	0.0313 (17)	0.0440 (19)	0.0266 (16)	−0.0033 (14)	0.0050 (13)	−0.0089 (14)
C16	0.0273 (16)	0.0286 (16)	0.0353 (17)	0.0005 (13)	0.0120 (13)	−0.0029 (13)
C17	0.0202 (14)	0.0224 (14)	0.0318 (16)	−0.0013 (11)	0.0025 (12)	0.0081 (12)
C18	0.0249 (15)	0.0188 (14)	0.0242 (14)	−0.0032 (11)	0.0001 (11)	0.0017 (11)
C19	0.0190 (13)	0.0237 (14)	0.0138 (12)	0.0000 (11)	0.0033 (10)	0.0008 (11)
C20	0.0255 (15)	0.0239 (15)	0.0201 (13)	0.0029 (12)	0.0014 (11)	−0.0003 (11)
C21	0.0237 (15)	0.0321 (16)	0.0218 (14)	0.0036 (12)	−0.0025 (11)	0.0010 (12)
C22	0.0218 (14)	0.0307 (15)	0.0186 (13)	−0.0038 (12)	−0.0001 (11)	−0.0013 (12)
C23	0.0216 (14)	0.0239 (14)	0.0156 (12)	−0.0020 (11)	0.0028 (11)	0.0015 (11)
C24	0.0173 (13)	0.0239 (14)	0.0154 (13)	−0.0011 (11)	0.0036 (10)	0.0032 (10)
C25	0.0214 (14)	0.0277 (15)	0.0229 (14)	−0.0038 (12)	0.0023 (11)	0.0055 (12)
C26	0.0287 (16)	0.0289 (16)	0.0258 (15)	−0.0029 (13)	0.0026 (12)	−0.0022 (13)
C27	0.0229 (15)	0.0259 (16)	0.0203 (14)	−0.0032 (12)	−0.0061 (11)	0.0051 (12)
C28	0.0262 (15)	0.0281 (15)	0.0165 (13)	0.0028 (13)	−0.0050 (11)	−0.0001 (12)
C29	0.0249 (14)	0.0165 (13)	0.0245 (15)	−0.0009 (11)	0.0044 (12)	−0.0011 (11)

### Tricarbonyl[1,3-diphenyl-6-(3-vinylphenyl)fulvene]chromium(0) (II) Geometric parameters (Å, º)

**Table:**

Cr1—C27	1.852 (3)	C10—H10	0.95
Cr1—C29	1.861 (3)	C11—C12	1.382 (4)
Cr1—C28	1.862 (3)	C11—H11	0.95
Cr1—C5	2.066 (3)	C12—H12	0.95
Cr1—C4	2.162 (3)	C13—C18	1.393 (4)
Cr1—C1	2.186 (2)	C13—C14	1.393 (4)
Cr1—C2	2.243 (3)	C14—C15	1.383 (4)
Cr1—C3	2.251 (3)	C14—H14	0.95
Cr1—C6	2.448 (3)	C15—C16	1.386 (4)
O1—C27	1.160 (3)	C15—H15	0.95
O2—C28	1.154 (3)	C16—C17	1.388 (4)
O3—C29	1.160 (3)	C16—H16	0.95
C1—C2	1.403 (4)	C17—C18	1.387 (4)
C1—C5	1.468 (4)	C17—H17	0.95
C1—C7	1.480 (4)	C18—H18	0.95
C2—C3	1.444 (4)	C19—C24	1.393 (4)
C2—H2	0.95	C19—C20	1.403 (4)
C3—C4	1.406 (4)	C20—C21	1.389 (4)
C3—C13	1.487 (4)	C20—H20	0.95
C4—C5	1.449 (4)	C21—C22	1.388 (4)
C4—H4	0.95	C21—H21	0.95
C5—C6	1.412 (4)	C22—C23	1.398 (4)
C6—C19	1.473 (4)	C22—H22	0.95
C6—H6	0.95	C23—C24	1.396 (4)
C7—C8	1.392 (4)	C23—C25	1.475 (4)
C7—C12	1.402 (4)	C24—H24	0.95
C8—C9	1.389 (4)	C25—C26	1.323 (4)
C8—H8	0.95	C25—H25	0.95
C9—C10	1.385 (4)	C26—H26A	0.95
C9—H9	0.95	C26—H26B	0.95
C10—C11	1.379 (4)		
			
C27—Cr1—C29	95.94 (11)	C19—C6—Cr1	121.37 (17)
C27—Cr1—C28	86.42 (12)	C5—C6—H6	117.7
C29—Cr1—C28	87.80 (11)	C19—C6—H6	117.7
C27—Cr1—C5	111.83 (11)	Cr1—C6—H6	91.1
C29—Cr1—C5	109.29 (11)	C8—C7—C12	118.7 (2)
C28—Cr1—C5	152.68 (11)	C8—C7—C1	121.9 (2)
C27—Cr1—C4	151.80 (11)	C12—C7—C1	119.4 (2)
C29—Cr1—C4	94.35 (10)	C9—C8—C7	120.1 (3)
C28—Cr1—C4	120.20 (11)	C9—C8—H8	119.9
C5—Cr1—C4	40.01 (10)	C7—C8—H8	119.9
C27—Cr1—C1	93.61 (11)	C10—C9—C8	120.6 (3)
C29—Cr1—C1	149.12 (11)	C10—C9—H9	119.7
C28—Cr1—C1	122.13 (11)	C8—C9—H9	119.7
C5—Cr1—C1	40.28 (10)	C11—C10—C9	119.6 (3)
C4—Cr1—C1	65.29 (9)	C11—C10—H10	120.2
C27—Cr1—C2	111.59 (11)	C9—C10—H10	120.2
C29—Cr1—C2	152.22 (11)	C10—C11—C12	120.4 (3)
C28—Cr1—C2	90.19 (11)	C10—C11—H11	119.8
C5—Cr1—C2	64.64 (10)	C12—C11—H11	119.8
C4—Cr1—C2	63.07 (10)	C11—C12—C7	120.5 (3)
C1—Cr1—C2	36.92 (9)	C11—C12—H12	119.7
C27—Cr1—C3	148.76 (11)	C7—C12—H12	119.7
C29—Cr1—C3	114.76 (10)	C18—C13—C14	118.6 (2)
C28—Cr1—C3	89.04 (11)	C18—C13—C3	120.4 (2)
C5—Cr1—C3	64.69 (10)	C14—C13—C3	120.7 (2)
C4—Cr1—C3	37.07 (9)	C15—C14—C13	120.9 (3)
C1—Cr1—C3	63.16 (9)	C15—C14—H14	119.5
C2—Cr1—C3	37.47 (9)	C13—C14—H14	119.5
C27—Cr1—C6	89.04 (11)	C14—C15—C16	120.1 (3)
C29—Cr1—C6	86.20 (10)	C14—C15—H15	119.9
C28—Cr1—C6	172.06 (10)	C16—C15—H15	119.9
C5—Cr1—C6	35.18 (9)	C15—C16—C17	119.6 (3)
C4—Cr1—C6	65.52 (9)	C15—C16—H16	120.2
C1—Cr1—C6	64.64 (9)	C17—C16—H16	120.2
C2—Cr1—C6	97.54 (9)	C18—C17—C16	120.2 (3)
C3—Cr1—C6	98.19 (9)	C18—C17—H17	119.9
C2—C1—C5	107.0 (2)	C16—C17—H17	119.9
C2—C1—C7	128.3 (2)	C17—C18—C13	120.6 (3)
C5—C1—C7	124.7 (2)	C17—C18—H18	119.7
C2—C1—Cr1	73.76 (14)	C13—C18—H18	119.7
C5—C1—Cr1	65.45 (13)	C24—C19—C20	118.6 (2)
C7—C1—Cr1	126.16 (18)	C24—C19—C6	120.8 (2)
C1—C2—C3	109.5 (2)	C20—C19—C6	120.5 (2)
C1—C2—Cr1	69.33 (14)	C21—C20—C19	120.3 (3)
C3—C2—Cr1	71.58 (14)	C21—C20—H20	119.9
C1—C2—H2	125.3	C19—C20—H20	119.9
C3—C2—H2	125.3	C22—C21—C20	120.3 (3)
Cr1—C2—H2	125.4	C22—C21—H21	119.8
C4—C3—C2	108.0 (2)	C20—C21—H21	119.8
C4—C3—C13	125.2 (2)	C21—C22—C23	120.4 (3)
C2—C3—C13	126.5 (2)	C21—C22—H22	119.8
C4—C3—Cr1	68.02 (14)	C23—C22—H22	119.8
C2—C3—Cr1	70.95 (14)	C24—C23—C22	118.7 (2)
C13—C3—Cr1	131.03 (18)	C24—C23—C25	118.6 (2)
C3—C4—C5	108.4 (2)	C22—C23—C25	122.7 (2)
C3—C4—Cr1	74.91 (15)	C19—C24—C23	121.6 (2)
C5—C4—Cr1	66.40 (14)	C19—C24—H24	119.2
C3—C4—H4	125.8	C23—C24—H24	119.2
C5—C4—H4	125.8	C26—C25—C23	125.5 (3)
Cr1—C4—H4	124.4	C26—C25—H25	117.3
C6—C5—C4	122.3 (2)	C23—C25—H25	117.3
C6—C5—C1	119.4 (2)	C25—C26—H26A	120.0
C4—C5—C1	107.0 (2)	C25—C26—H26B	120.0
C6—C5—Cr1	87.36 (16)	H26A—C26—H26B	120.0
C4—C5—Cr1	73.58 (14)	O1—C27—Cr1	178.8 (2)
C1—C5—Cr1	74.26 (14)	O2—C28—Cr1	179.1 (2)
C5—C6—C19	124.5 (2)	O3—C29—Cr1	178.9 (2)
C5—C6—Cr1	57.46 (14)		

### Tricarbonyl[1,3-diphenyl-6-(pyren-1-yl)fulvene]chromium(0) (III) Crystal data

**Table:**

[Cr(C_34_H_22_)(CO)_3_]	*Z* = 2
*M**_r_* = 566.54	*F*(000) = 584
Triclinic, *P*1	*D*_x_ = 1.421 Mg m^−^^3^
*a* = 8.6342 (12) Å	Mo *K*α radiation, λ = 0.71073 Å
*b* = 9.4070 (13) Å	Cell parameters from 9972 reflections
*c* = 17.379 (3) Å	θ = 2.3–27.2°
α = 82.988 (2)°	µ = 0.47 mm^−^^1^
β = 86.340 (2)°	*T* = 100 K
γ = 70.992 (2)°	Ractangular prism, translucent red
*V* = 1324.2 (3) Å^3^	0.27 × 0.20 × 0.11 mm

### Tricarbonyl[1,3-diphenyl-6-(pyren-1-yl)fulvene]chromium(0) (III) Data collection

**Table:**

Bruker SMART APEX CCD diffractometer	5892 independent reflections
Radiation source: fine focus sealed tube	5241 reflections with *I* > 2σ(*I*)
Graphite monochromator	*R*_int_ = 0.029
Detector resolution: 8.3333 pixels mm^-1^	θ_max_ = 27.3°, θ_min_ = 2.3°
ω Scans scans	*h* = −11→11
Absorption correction: multi-scan (SADABS; Bruker, 2017)	*k* = −12→12
*T*_min_ = 0.88, *T*_max_ = 0.95	*l* = −22→22
25620 measured reflections	

### Tricarbonyl[1,3-diphenyl-6-(pyren-1-yl)fulvene]chromium(0) (III) Refinement

**Table:**

Refinement on *F*^2^	0 restraints
Least-squares matrix: full	Hydrogen site location: inferred from neighbouring sites
*R*[*F*^2^ > 2σ(*F*^2^)] = 0.036	H-atom parameters constrained
*wR*(*F*^2^) = 0.098	*w* = 1/[σ^2^(*F*_o_^2^) + (0.0465*P*)^2^ + 0.9304*P*] where *P* = (*F*_o_^2^ + 2*F*_c_^2^)/3
*S* = 1.05	(Δ/σ)_max_ = 0.001
5892 reflections	Δρ_max_ = 0.48 e Å^−^^3^
370 parameters	Δρ_min_ = −0.38 e Å^−^^3^

### Tricarbonyl[1,3-diphenyl-6-(pyren-1-yl)fulvene]chromium(0) (III) Special details

**Table:**

Geometry. All esds (except the esd in the dihedral angle between two l.s. planes) are estimated using the full covariance matrix. The cell esds are taken into account individually in the estimation of esds in distances, angles and torsion angles; correlations between esds in cell parameters are only used when they are defined by crystal symmetry. An approximate (isotropic) treatment of cell esds is used for estimating esds involving l.s. planes.

### Tricarbonyl[1,3-diphenyl-6-(pyren-1-yl)fulvene]chromium(0) (III) Fractional atomic coordinates and isotropic or equivalent isotropic displacement parameters (Å^2^)

**Table:**

	*x*	*y*	*z*	*U*_iso_*/*U*_eq_	
Cr1	0.79583 (3)	0.56080 (3)	0.17877 (2)	0.01584 (9)	
O1	1.12873 (17)	0.45249 (18)	0.25019 (9)	0.0378 (3)	
O2	0.94096 (18)	0.74249 (16)	0.05740 (8)	0.0333 (3)	
O3	0.63615 (17)	0.84788 (14)	0.25289 (7)	0.0288 (3)	
C1	0.79878 (19)	0.33225 (18)	0.16578 (9)	0.0165 (3)	
C2	0.7743 (2)	0.41273 (18)	0.09148 (9)	0.0178 (3)	
H2	0.845365	0.387665	0.047645	0.021*	
C3	0.6249 (2)	0.53916 (18)	0.09196 (9)	0.0186 (3)	
C4	0.55759 (19)	0.53829 (18)	0.16815 (9)	0.0175 (3)	
H4	0.457664	0.60765	0.1849	0.021*	
C5	0.66654 (19)	0.41368 (18)	0.21673 (9)	0.0164 (3)	
C6	0.70058 (19)	0.43062 (18)	0.29293 (9)	0.0161 (3)	
H6	0.808633	0.381687	0.310928	0.019*	
C7	0.9293 (2)	0.18919 (18)	0.18909 (9)	0.0175 (3)	
C8	0.8958 (2)	0.0844 (2)	0.24615 (10)	0.0228 (4)	
H8	0.789757	0.106144	0.269969	0.027*	
C9	1.0163 (2)	−0.0514 (2)	0.26838 (11)	0.0283 (4)	
H9	0.992017	−0.121957	0.307175	0.034*	
C10	1.1718 (2)	−0.0844 (2)	0.23422 (11)	0.0283 (4)	
H10	1.25462	−0.176638	0.250145	0.034*	
C11	1.2060 (2)	0.0184 (2)	0.17637 (11)	0.0255 (4)	
H11	1.31197	−0.004279	0.15235	0.031*	
C12	1.0856 (2)	0.15373 (19)	0.15384 (10)	0.0211 (3)	
H12	1.109467	0.223	0.114144	0.025*	
C13	0.5535 (2)	0.64959 (19)	0.02498 (10)	0.0207 (3)	
C14	0.6312 (2)	0.6415 (2)	−0.04781 (10)	0.0250 (4)	
H14	0.731533	0.563223	−0.055193	0.03*	
C15	0.5627 (2)	0.7474 (2)	−0.10996 (11)	0.0302 (4)	
H15	0.615891	0.740608	−0.159585	0.036*	
C16	0.4170 (3)	0.8628 (2)	−0.09937 (12)	0.0332 (5)	
H16	0.371636	0.936123	−0.141516	0.04*	
C17	0.3376 (3)	0.8714 (2)	−0.02766 (12)	0.0347 (5)	
H17	0.237592	0.950215	−0.020503	0.042*	
C18	0.4045 (2)	0.7643 (2)	0.03405 (11)	0.0287 (4)	
H18	0.348324	0.769108	0.082958	0.034*	
C19	0.57727 (19)	0.52021 (18)	0.34598 (9)	0.0155 (3)	
C20	0.41212 (19)	0.53205 (18)	0.33991 (9)	0.0172 (3)	
H20	0.382838	0.479264	0.302989	0.021*	
C21	0.29053 (19)	0.61925 (18)	0.38672 (9)	0.0179 (3)	
H21	0.1798	0.62484	0.381434	0.021*	
C22	0.32897 (19)	0.69884 (18)	0.44146 (9)	0.0166 (3)	
C23	0.2054 (2)	0.79626 (19)	0.48839 (10)	0.0198 (3)	
H23	0.093226	0.809317	0.481207	0.024*	
C24	0.2452 (2)	0.86931 (19)	0.54228 (10)	0.0218 (3)	
H24	0.160632	0.933498	0.571833	0.026*	
C25	0.4132 (2)	0.85192 (18)	0.55585 (9)	0.0193 (3)	
C26	0.4580 (2)	0.9247 (2)	0.61209 (10)	0.0235 (4)	
H26	0.374925	0.990061	0.641848	0.028*	
C27	0.6212 (2)	0.9035 (2)	0.62527 (10)	0.0249 (4)	
H27	0.648894	0.951382	0.664939	0.03*	
C28	0.7445 (2)	0.8122 (2)	0.58053 (10)	0.0226 (4)	
H28	0.856009	0.798161	0.589879	0.027*	
C29	0.7055 (2)	0.74059 (18)	0.52168 (9)	0.0180 (3)	
C30	0.8282 (2)	0.65028 (19)	0.47246 (10)	0.0192 (3)	
H30	0.940111	0.638468	0.479502	0.023*	
C31	0.78892 (19)	0.58098 (18)	0.41591 (9)	0.0180 (3)	
H31	0.873582	0.523808	0.383688	0.022*	
C32	0.62204 (19)	0.59243 (18)	0.40390 (9)	0.0157 (3)	
C33	0.49676 (19)	0.68261 (17)	0.45165 (9)	0.0146 (3)	
C34	0.5386 (2)	0.75816 (18)	0.50973 (9)	0.0166 (3)	
C35	1.0005 (2)	0.4960 (2)	0.22347 (10)	0.0230 (4)	
C36	0.8848 (2)	0.67370 (19)	0.10356 (10)	0.0219 (3)	
C37	0.7019 (2)	0.73753 (19)	0.22550 (9)	0.0198 (3)	

### Tricarbonyl[1,3-diphenyl-6-(pyren-1-yl)fulvene]chromium(0) (III) Atomic displacement parameters (Å^2^)

**Table:**

	*U*^11^	*U*^22^	*U*^33^	*U*^12^	*U*^13^	*U*^23^
Cr1	0.01724 (14)	0.01592 (14)	0.01408 (14)	−0.00495 (10)	−0.00016 (10)	−0.00165 (10)
O1	0.0248 (7)	0.0462 (9)	0.0427 (9)	−0.0099 (6)	−0.0109 (6)	−0.0053 (7)
O2	0.0394 (8)	0.0318 (7)	0.0289 (7)	−0.0148 (6)	0.0093 (6)	0.0003 (6)
O3	0.0414 (8)	0.0194 (6)	0.0235 (6)	−0.0066 (6)	0.0044 (6)	−0.0053 (5)
C1	0.0180 (7)	0.0164 (8)	0.0155 (7)	−0.0060 (6)	0.0008 (6)	−0.0028 (6)
C2	0.0214 (8)	0.0176 (8)	0.0152 (7)	−0.0072 (6)	0.0005 (6)	−0.0035 (6)
C3	0.0214 (8)	0.0181 (8)	0.0175 (8)	−0.0071 (6)	−0.0039 (6)	−0.0020 (6)
C4	0.0167 (7)	0.0180 (8)	0.0177 (8)	−0.0053 (6)	−0.0018 (6)	−0.0022 (6)
C5	0.0169 (7)	0.0156 (7)	0.0172 (8)	−0.0065 (6)	0.0005 (6)	−0.0010 (6)
C6	0.0150 (7)	0.0166 (8)	0.0164 (7)	−0.0052 (6)	0.0000 (6)	−0.0007 (6)
C7	0.0203 (8)	0.0171 (8)	0.0143 (7)	−0.0038 (6)	−0.0009 (6)	−0.0042 (6)
C8	0.0237 (8)	0.0211 (9)	0.0205 (8)	−0.0037 (7)	0.0032 (7)	−0.0018 (7)
C9	0.0357 (10)	0.0207 (9)	0.0224 (9)	−0.0037 (8)	0.0029 (8)	0.0041 (7)
C10	0.0298 (9)	0.0178 (8)	0.0285 (9)	0.0039 (7)	−0.0011 (8)	−0.0014 (7)
C11	0.0229 (8)	0.0226 (9)	0.0275 (9)	−0.0022 (7)	0.0043 (7)	−0.0061 (7)
C12	0.0235 (8)	0.0191 (8)	0.0197 (8)	−0.0059 (7)	0.0028 (6)	−0.0022 (6)
C13	0.0257 (8)	0.0193 (8)	0.0190 (8)	−0.0097 (7)	−0.0062 (7)	0.0000 (6)
C14	0.0265 (9)	0.0295 (9)	0.0211 (8)	−0.0131 (7)	−0.0051 (7)	0.0031 (7)
C15	0.0351 (10)	0.0401 (11)	0.0215 (9)	−0.0231 (9)	−0.0073 (8)	0.0070 (8)
C16	0.0427 (11)	0.0287 (10)	0.0313 (10)	−0.0178 (9)	−0.0192 (9)	0.0122 (8)
C17	0.0402 (11)	0.0226 (9)	0.0365 (11)	−0.0022 (8)	−0.0155 (9)	0.0006 (8)
C18	0.0368 (10)	0.0226 (9)	0.0229 (9)	−0.0033 (8)	−0.0061 (8)	−0.0027 (7)
C19	0.0167 (7)	0.0149 (7)	0.0131 (7)	−0.0036 (6)	−0.0004 (6)	0.0013 (6)
C20	0.0183 (8)	0.0180 (8)	0.0155 (7)	−0.0069 (6)	−0.0015 (6)	0.0007 (6)
C21	0.0148 (7)	0.0191 (8)	0.0184 (8)	−0.0053 (6)	−0.0010 (6)	0.0033 (6)
C22	0.0169 (7)	0.0149 (7)	0.0159 (7)	−0.0042 (6)	0.0016 (6)	0.0022 (6)
C23	0.0160 (7)	0.0194 (8)	0.0208 (8)	−0.0034 (6)	0.0026 (6)	0.0020 (6)
C24	0.0218 (8)	0.0184 (8)	0.0214 (8)	−0.0033 (7)	0.0066 (6)	−0.0006 (6)
C25	0.0246 (8)	0.0165 (8)	0.0157 (8)	−0.0065 (6)	0.0028 (6)	0.0007 (6)
C26	0.0315 (9)	0.0209 (8)	0.0182 (8)	−0.0090 (7)	0.0064 (7)	−0.0044 (7)
C27	0.0357 (10)	0.0266 (9)	0.0170 (8)	−0.0156 (8)	0.0019 (7)	−0.0051 (7)
C28	0.0260 (9)	0.0260 (9)	0.0186 (8)	−0.0124 (7)	−0.0025 (7)	−0.0003 (7)
C29	0.0224 (8)	0.0171 (8)	0.0144 (7)	−0.0076 (6)	−0.0004 (6)	0.0015 (6)
C30	0.0163 (7)	0.0214 (8)	0.0194 (8)	−0.0065 (6)	−0.0015 (6)	0.0011 (6)
C31	0.0162 (7)	0.0192 (8)	0.0167 (8)	−0.0037 (6)	0.0016 (6)	−0.0012 (6)
C32	0.0168 (7)	0.0154 (7)	0.0130 (7)	−0.0040 (6)	−0.0007 (6)	0.0022 (6)
C33	0.0174 (7)	0.0126 (7)	0.0127 (7)	−0.0046 (6)	0.0003 (6)	0.0027 (6)
C34	0.0202 (8)	0.0152 (7)	0.0132 (7)	−0.0052 (6)	0.0003 (6)	0.0019 (6)
C35	0.0256 (9)	0.0235 (9)	0.0220 (8)	−0.0101 (7)	0.0004 (7)	−0.0044 (7)
C36	0.0233 (8)	0.0210 (8)	0.0210 (8)	−0.0061 (7)	0.0008 (7)	−0.0045 (7)
C37	0.0247 (8)	0.0202 (8)	0.0154 (8)	−0.0096 (7)	−0.0004 (6)	0.0013 (6)

### Tricarbonyl[1,3-diphenyl-6-(pyren-1-yl)fulvene]chromium(0) (III) Geometric parameters (Å, º)

**Table:**

Cr1—C37	1.8570 (18)	C14—C15	1.394 (2)
Cr1—C35	1.8589 (18)	C14—H14	0.95
Cr1—C36	1.8684 (18)	C15—C16	1.388 (3)
Cr1—C5	2.0704 (16)	C15—H15	0.95
Cr1—C4	2.1569 (16)	C16—C17	1.382 (3)
Cr1—C1	2.1809 (16)	C16—H16	0.95
Cr1—C2	2.2353 (16)	C17—C18	1.391 (3)
Cr1—C3	2.2477 (16)	C17—H17	0.95
Cr1—C6	2.4428 (16)	C18—H18	0.95
O1—C35	1.155 (2)	C19—C20	1.403 (2)
O2—C36	1.151 (2)	C19—C32	1.418 (2)
O3—C37	1.153 (2)	C20—C21	1.389 (2)
C1—C2	1.406 (2)	C20—H20	0.95
C1—C5	1.463 (2)	C21—C22	1.397 (2)
C1—C7	1.477 (2)	C21—H21	0.95
C2—C3	1.443 (2)	C22—C33	1.427 (2)
C2—H2	0.95	C22—C23	1.442 (2)
C3—C4	1.412 (2)	C23—C24	1.349 (3)
C3—C13	1.481 (2)	C23—H23	0.95
C4—C5	1.454 (2)	C24—C25	1.437 (2)
C4—H4	0.95	C24—H24	0.95
C5—C6	1.413 (2)	C25—C26	1.399 (2)
C6—C19	1.474 (2)	C25—C34	1.425 (2)
C6—H6	0.95	C26—C27	1.388 (3)
C7—C8	1.396 (2)	C26—H26	0.95
C7—C12	1.400 (2)	C27—C28	1.392 (3)
C8—C9	1.388 (2)	C27—H27	0.95
C8—H8	0.95	C28—C29	1.404 (2)
C9—C10	1.387 (3)	C28—H28	0.95
C9—H9	0.95	C29—C34	1.423 (2)
C10—C11	1.394 (3)	C29—C30	1.432 (2)
C10—H10	0.95	C30—C31	1.360 (2)
C11—C12	1.387 (2)	C30—H30	0.95
C11—H11	0.95	C31—C32	1.436 (2)
C12—H12	0.95	C31—H31	0.95
C13—C14	1.392 (2)	C32—C33	1.428 (2)
C13—C18	1.398 (3)	C33—C34	1.429 (2)
			
C37—Cr1—C35	99.16 (8)	C8—C9—H9	119.8
C37—Cr1—C36	87.29 (7)	C9—C10—C11	119.63 (16)
C35—Cr1—C36	86.01 (8)	C9—C10—H10	120.2
C37—Cr1—C5	108.10 (7)	C11—C10—H10	120.2
C35—Cr1—C5	110.57 (7)	C12—C11—C10	120.12 (17)
C36—Cr1—C5	154.47 (7)	C12—C11—H11	119.9
C37—Cr1—C4	90.69 (7)	C10—C11—H11	119.9
C35—Cr1—C4	150.55 (7)	C11—C12—C7	120.59 (16)
C36—Cr1—C4	122.35 (7)	C11—C12—H12	119.7
C5—Cr1—C4	40.16 (6)	C7—C12—H12	119.7
C37—Cr1—C1	148.22 (7)	C14—C13—C18	118.68 (16)
C35—Cr1—C1	93.85 (7)	C14—C13—C3	121.37 (16)
C36—Cr1—C1	122.63 (7)	C18—C13—C3	119.95 (16)
C5—Cr1—C1	40.16 (6)	C13—C14—C15	120.46 (18)
C4—Cr1—C1	65.28 (6)	C13—C14—H14	119.8
C37—Cr1—C2	147.66 (7)	C15—C14—H14	119.8
C35—Cr1—C2	112.97 (7)	C16—C15—C14	120.04 (18)
C36—Cr1—C2	91.17 (7)	C16—C15—H15	120.0
C5—Cr1—C2	64.82 (6)	C14—C15—H15	120.0
C4—Cr1—C2	63.18 (6)	C17—C16—C15	120.14 (17)
C1—Cr1—C2	37.11 (6)	C17—C16—H16	119.9
C37—Cr1—C3	110.15 (7)	C15—C16—H16	119.9
C35—Cr1—C3	150.34 (7)	C16—C17—C18	119.82 (19)
C36—Cr1—C3	90.74 (7)	C16—C17—H17	120.1
C5—Cr1—C3	65.12 (6)	C18—C17—H17	120.1
C4—Cr1—C3	37.32 (6)	C17—C18—C13	120.82 (18)
C1—Cr1—C3	63.42 (6)	C17—C18—H18	119.6
C2—Cr1—C3	37.54 (6)	C13—C18—H18	119.6
C37—Cr1—C6	86.76 (6)	C20—C19—C32	119.24 (14)
C35—Cr1—C6	87.32 (7)	C20—C19—C6	119.21 (14)
C36—Cr1—C6	170.24 (7)	C32—C19—C6	121.54 (14)
C5—Cr1—C6	35.29 (6)	C21—C20—C19	121.44 (15)
C4—Cr1—C6	65.47 (6)	C21—C20—H20	119.3
C1—Cr1—C6	64.92 (6)	C19—C20—H20	119.3
C2—Cr1—C6	97.98 (6)	C20—C21—C22	120.85 (15)
C3—Cr1—C6	98.57 (6)	C20—C21—H21	119.6
C2—C1—C5	107.34 (14)	C22—C21—H21	119.6
C2—C1—C7	127.29 (14)	C21—C22—C33	118.86 (14)
C5—C1—C7	125.35 (14)	C21—C22—C23	122.43 (15)
C2—C1—Cr1	73.55 (9)	C33—C22—C23	118.71 (15)
C5—C1—Cr1	65.85 (8)	C24—C23—C22	121.55 (15)
C7—C1—Cr1	127.16 (11)	C24—C23—H23	119.2
C1—C2—C3	109.65 (14)	C22—C23—H23	119.2
C1—C2—Cr1	69.34 (9)	C23—C24—C25	121.28 (15)
C3—C2—Cr1	71.70 (9)	C23—C24—H24	119.4
C1—C2—H2	125.2	C25—C24—H24	119.4
C3—C2—H2	125.2	C26—C25—C34	118.90 (16)
Cr1—C2—H2	125.4	C26—C25—C24	122.42 (16)
C4—C3—C2	107.49 (14)	C34—C25—C24	118.68 (15)
C4—C3—C13	125.94 (15)	C27—C26—C25	121.33 (16)
C2—C3—C13	126.57 (15)	C27—C26—H26	119.3
C4—C3—Cr1	67.85 (9)	C25—C26—H26	119.3
C2—C3—Cr1	70.76 (9)	C26—C27—C28	120.19 (16)
C13—C3—Cr1	126.97 (11)	C26—C27—H27	119.9
C3—C4—C5	108.71 (14)	C28—C27—H27	119.9
C3—C4—Cr1	74.84 (9)	C27—C28—C29	120.57 (16)
C5—C4—Cr1	66.72 (9)	C27—C28—H28	119.7
C3—C4—H4	125.6	C29—C28—H28	119.7
C5—C4—H4	125.6	C28—C29—C34	119.36 (15)
Cr1—C4—H4	124.3	C28—C29—C30	122.37 (15)
C6—C5—C4	121.28 (14)	C34—C29—C30	118.27 (15)
C6—C5—C1	120.01 (14)	C31—C30—C29	121.80 (15)
C4—C5—C1	106.66 (13)	C31—C30—H30	119.1
C6—C5—Cr1	86.90 (10)	C29—C30—H30	119.1
C4—C5—Cr1	73.12 (9)	C30—C31—C32	121.37 (15)
C1—C5—Cr1	73.99 (9)	C30—C31—H31	119.3
C5—C6—C19	123.47 (14)	C32—C31—H31	119.3
C5—C6—Cr1	57.81 (8)	C19—C32—C33	119.11 (14)
C19—C6—Cr1	119.16 (10)	C19—C32—C31	122.71 (14)
C5—C6—H6	118.3	C33—C32—C31	118.17 (14)
C19—C6—H6	118.3	C22—C33—C32	120.32 (14)
Cr1—C6—H6	92.8	C22—C33—C34	119.51 (14)
C8—C7—C12	118.75 (15)	C32—C33—C34	120.17 (14)
C8—C7—C1	119.66 (15)	C29—C34—C25	119.59 (15)
C12—C7—C1	121.57 (15)	C29—C34—C33	120.18 (14)
C9—C8—C7	120.59 (16)	C25—C34—C33	120.23 (15)
C9—C8—H8	119.7	O1—C35—Cr1	178.26 (16)
C7—C8—H8	119.7	O2—C36—Cr1	179.42 (17)
C10—C9—C8	120.31 (17)	O3—C37—Cr1	176.49 (15)
C10—C9—H9	119.8		
